# Exploring the experiences and perceptions of disease‐modifying treatments among Canadians affected by dementia

**DOI:** 10.1002/alz70858_106050

**Published:** 2025-12-26

**Authors:** Sarah A. Wu, Adam Morrison, Carolina Caputo Oliveira, Cari Randa‐Beaulieu, Heather A. Cooke

**Affiliations:** ^1^ Alzheimer Society of B.C., Vancouver, BC, Canada; ^2^ Alzheimer Society of Ontario, Toronto, ON, Canada

## Abstract

**Background:**

Discourse on disease‐modifying treatments (DMTs) for dementia has primarily focused on drug development, health system readiness and health care professional perspectives. There is a paucity of research focused on public perceptions held by Canadians towards these treatments. Focus groups were conducted in British Columbia and Ontario, Canada with people affected by dementia to gain an in‐depth understanding of their experiences with and perceptions of DMTs.

**Method:**

Between February and December 2024, the Alzheimer Society of B.C. (ASBC) and the Alzheimer Society of Ontario (ASO) held a series of separate focus groups. ASBC conducted four online focus groups with eight persons living with dementia and nine care partners, none of whom were enrolled in DMT clinical trials. ASO conducted three in‐person and two online focus groups with people living with dementia (*n* = 12) and their care partners (*n* = 11) enrolled in DMT clinical trials. Focus groups ranged from 90‐120 minutes and consisted of questions exploring treatments, risk tolerance and the role of our organizations to support patient navigation for DMTs. Data were analyzed using thematic analysis.

**Result:**

Focus group participant experiences ranged depending on disease progression. Four themes were identified. The first, equitable access to DMTs, highlighted concerns around financial hardship, geographic limitations and the critical importance of care partner support. The second, risk tolerance, underscored differences in risk acceptance among people living with dementia and their care partners, and the variation across disease stage. The third, clear and accessible information, focused on the need for comprehensive, transparent and easy‐to‐understand material to ensure informed DMT decision‐making. The final theme, DMTs as a source of hope, acknowledged the motivational role of clinical trials and DMT approvals symbolizing innovation and the potential for an eventual cure. ASBC and ASO were identified as trustworthy and important sources of support for DMT navigation.

**Conclusion:**

Findings identify priority areas for people affected by dementia once DMTs become available in Canada. Collaborating with people with lived experience in the development of DMT care pathways will help to ensure that treatment options are accessible, deliver meaningful benefits to extend quality of life and improve quality of care.

